# Hypercontractile cardiac phenotype in mice overexpressing the regulatory subunit PR72 of protein phosphatase 2A

**DOI:** 10.3389/fcvm.2023.1239555

**Published:** 2023-10-06

**Authors:** Julius R. Herting, Jule H. König, Katarina Hadova, Alexander Heinick, Frank U. Müller, Paul Pauls, Matthias D. Seidl, Carolina Soppa, Uwe Kirchhefer

**Affiliations:** ^1^ Institut für Pharmakologie und Toxikologie, Universitätsklinikum Münster, Universität Münster, Münster, Germany; ^2^ Department of Pharmacology and Toxicology, Faculty of Pharmacy, Comenius University in Bratislava, Bratislava, Slovakia

**Keywords:** protein phosphatase 2A, regulatory B'' subunit PPP2R3A/PR72, contractility, Ca^2+^ handling, protein phosphorylation, Na^+^/Ca^2+^ exchanger

## Abstract

**Background:**

The activity, localization, and substrate specificity of the protein phosphatase 2A (PP2A) heterotrimer are controlled by various regulatory B subunits. PR72 belongs to the B'' gene family and has been shown to be upregulated in human heart failure. However, little is known about the functions of PR72 in the myocardium.

**Methods:**

To address this issue, we generated a transgenic mouse model with heart-specific overexpression of PP2A-PR72. Biochemical and physiological methods were used to determine contractility, Ca^2+^ cycling parameters, and protein phosphorylation.

**Results:**

A 2.5-fold increase in PR72 expression resulted in moderate cardiac hypertrophy. Maximal ventricular pressure was increased in catheterized transgenic mice (TG) compared to wild-type (WT) littermates. This was accompanied by an increased shortening of sarcomere length and faster relaxation at the single-cell level in TG. In parallel with these findings, the peak amplitude of Ca^2+^ transients was increased, and the decay in intracellular Ca^2+^ levels was shortened in TG compared to WT. The changes in Ca^2+^ cycling in TG were also evident from an increase in the full duration and width at half maximum of Ca^2+^ sparks. Consistent with the contractile data, phosphorylation of phospholamban at threonine-17 was higher in TG hearts. The lower expression of the Na^+^/Ca^2+^ exchanger may also contribute to the hypercontractile state in transgenic myocardium.

**Conclusion:**

Our results suggest that PP2A-PR72 plays an important role in regulating cardiac contractile function and Ca^2+^ cycling, indicating that the upregulation of PR72 in heart failure is an attempt to compensate functionally.

## Introduction

Protein phosphatase 2A (PP2A) is an enzyme that specifically removes phosphates from serine and threonine residues and is essential for various signaling pathways involved in cell development and growth (1, 2). PP2A is unique in its structure, composed of three subunits - catalytic C, scaffolding A, and a variable regulatory B subunit from four gene families that control subcellular localization, substrate specificity, and enzyme activity (3). Currently, 24 regulatory B subunits have been described and are divided into four families - PR55/B, PR61/B56/B', PR72/B'', and PR93/PR110/B''' based on their structure (4). The combination of these subunits can yield approximately 100 different trimeric ABC holoenzymes depending on the tissue-specific expression of PP2A subunits (1).

Studies on the tissue-specific expression of regulatory B subunits have shown that they are also expressed in the heart, where PP2A plays a major role in cardiac physiology (3). PP2A dephosphorylates ion channels, transporters, and myofilament proteins to modulate excitability, contractility, and metabolism of the myocardium (5). While numerous studies have been conducted on members of the regulatory B and B' families [for review see: (3)], few data exist on PR72/B''. PR72 is the *α*_2_ splice variant of the *PPP2R3A* gene and belongs to the PR72/B'' family, named after the molecular weight of the first identified member (6). Characteristically for this family and therefore also for PR72 are two conserved A subunit-binding domains and two Ca-binding EF-hand motifs (7, 8). Mutational studies on these EF-hand sites indicate that Ca^2+^ can influence the assembly and activity of the PR72-containing PP2A (8, 9). While one group detected a decrease in the activity of the tissue-derived purified PR72-containing PP2A trimer under increasing Ca^2+^ concentrations (8), in contrast, another group was able to measure an increase in enzyme activity for various phosphosubstrates under higher Ca^2+^ concentrations using an immunoprecipitated PP2A-PR72 holoenzyme (9). Thus, it can be speculated that alterations in myocardial Ca^2+^ homeostasis, as observed in heart failure, ischemic cardiomyopathy, and ventricular tachyarrhythmia [for review see (10, 11)], may also have implications for the functional state of PR72-associated PP2A activity.

Increased expression of PR72 at the mRNA and protein levels in human ischemic heart failure may reflect a detrimental effect (12). This hypothesis is supported by data on pulmonary fibroblasts that developed irreversible fibrosis after overexpression of PR72 (13). In contrast, silencing of the PPP2R3A gene was able to inhibit myocardial cell proliferation and promote cardiomyocyte apoptosis (14). Whether PP2A-PR72 exerts these effects directly or via specific interaction partners at targets yet to be determined remains unknown.

PR72 is found inside heart muscle cells at specific locations known as Z- and M-lines. This suggests that it has a structural role (12). Studies in zebrafish with a genetic deletion of PR72 have shown defects in cardiac development, including enlarged ventricular chambers and reduced cardiac function (15). The deletion of PR72 also affects the structure of the sarcomere, particularly the architecture of the Z-lines. In mice, a reduction in PR72 protein levels was associated with cardiac contractile dysfunction, impaired phospholamban phosphorylation and prolonged myocellular Ca^2+^ decay when the epidermal growth factor receptor (EGFR) was downregulated in cardiomyocytes (16). In contrast to the fibrotic effects, the functional data from these animal models suggest that the increased expression of PR72 in heart failure may represent a positive response to disease-related changes, attempting to compensate for contractile deficits.

To examine whether the twofold increase in PR72 expression observed in human heart failure functions as a compensatory mechanism to offset contractile dysfunction, we generated a transgenic mouse model that specifically overexpresses PP2A-PR72 in the heart. The level of overexpression in transgenic mice was designed to reflect the rise in PR72 protein expression observed in heart failure. This mouse model was used to study the effects on contractility, myocellular calcium handling, and phosphorylation of regulatory proteins.

## Materials and methods

### Generation of PR72-transgenic mice

Transgenic (TG) mice with heart-specific overexpression of murine PR72 were generated by the transgenic animal and genetic engineering models core facility at the University of Münster. The *α*MHC-PR72 transgene was injected into the pronuclei of fertilized FVB/N mouse eggs. Southern blotting was used to identify six transgenic founder mice, which were then cross-bred with wild-type (WT) mice. The founder mouse that produced TG offspring with the highest levels of overexpression was selected for breeding littermates for subsequent experiments. All experiments were performed on 20- to 24-week-old mice. Balanced proportions of females and males were always used and animals were bred on a FVB/N strain background. All animal handling and maintenance procedures were conducted in accordance with approved protocols by the animal welfare committee of the University of Munster and the LANUV (NRW, Germany; ID 81-02.04.2021.A151). These protocols also complied with the National Institutes of Health Guidelines for the Care and Use of Laboratory Animals.

### Isolation of cardiomyocytes

Isolation of ventricular cardiomyocytes followed an approved protocol (17). Mice were killed by cervical dislocation, and the heart was immediately removed. The cannulated heart was then connected to a modified Langendorff apparatus and retrogradely perfused for 5 min with a perfusion buffer (17) at a flow rate of 2.5 ml/min. For another 7.25 min, digestion was performed with an enzyme solution containing Liberase DH. The aorta and atria were separated from the heart. The remaining heart was minced in a 5 ml enzyme stop solution. After 10 min of incubation, the sedimented pellet was transferred into 10 ml of an enzyme stop solution, filtered through nylon gauze, and centrifuged at 500 rpm for 1 min. The cell pellet was then resuspended in 10 ml of perfusion buffer and the Ca^2+^ concentration was raised stepwise to 1 mM. Then, centrifugation was performed again for 1 min at 500 rpm, and the cell pellet was resuspended in perfusion buffer containing 1 mM CaCl_2_. Cardiomyocytes were kept at room temperature and used within 6 h of isolation.

### Immunofluorescence

Isolated mouse cardiomyocytes were immobilized by a 10 min incubation with a 4% formaldehyde/PBS solution, followed by a PBS wash, placed on poly-Lysine-coated slides to adhere for a 30-min incubation and permeabilized with a solution containing PBS and 0.2% Tergitol 15-S-9 (NeoFroxx GmbH) for 10 min. The cells were then incubated in a blocking solution consisting of 2% goat serum (Sigma Aldrich) and 1% bovine serum albumin (BSA; Sigma Aldrich) in PBS-T (PBS with 1% Tween 20) for 1 h. This was followed by treatment with an antibody against human PR72 (1:200, HPA-035829, Sigma Aldrich) in the blocking solution for 1 h. After thorough washing (typically three times for 10 min each in PBS), the cardiomyocytes were incubated for 1 h at room temperature with a secondary antibody, Alexa Fluor 488 (1:500, Alexa Fluor® 488 chicken anti-rabbit IgG, Invitrogen). After another washing step, the cells were blocked with unconjugated Fab fragments [100 µg/ml goat anti-mouse IgG (H + L) unconjugated; Dianova] for 1 h. Antibodies specific for GAPDH (1:300, AM4300, Thermo Fisher) or sarcomeric *α*-actinin (1:200, A7732, Sigma Aldrich) was then applied for 1 h, followed by washing and incubation for 1 h with a secondary antibody, Alexa Fluor 594 (1:500, Alexa Fluor® 594 goat anti-mouse IgG, Invitrogen). Fluorescence signals were acquired using a confocal laser scanning microscope (LSM 710; Carl Zeiss AG) equipped with laser lines at 488 nm and 543 nm, operated at 2% power with 16-fold line averaging and a 33-*μ*m pinhole. Imaging was performed with a Plan Neofluar 25x/0.8 objective.

### Cloning of expression vectors (cDNA, *α*MHC, RGS-6xHis)

See supplementary methods section.

### Culture of HEK293 cells

Cells were cultured (37°C and 5% CO_2_) in Dulbecco's modified Eagle's medium (DMEM), supplemented with FCS (10% v/v, Cytogen) and Penicillin Streptomycin (100 U/ml, Sigma-Aldrich). DMEM low glucose (Sigma-Aldrich) was used for HEK293 cells (ATCC).

### Pull-down assay

To investigate the protein-protein interaction of PR72 with the catalytic PP2A subunit, pull-down assays were performed. HEK293 cells were transiently transfected with the N-terminal RGS6xHis-tagged PR72 cDNA or the empty RGS6xHis-pcDNA3.1 vector as a control in 75-cm^2^ culture flasks. Xtreme gene-HP DNA (DNA ratio 1:1; Roche Diagnostics) was used for transfection according to the manufacturer's protocol. After 48 h, the cells were washed with PBS, scraped in 4 ml PBS, centrifuged at 100 × g and lysed with 600 µl lysis buffer (50 mM NaH_2_PO_4_, 300 mM NaCl, 1% NP40, pH 8.0, supplemented with protease and phosphatase inhibitors). Lysates were sonicated four times for 15 s, incubated on ice for 20 min and centrifuged at 21,000 × g for 10 min. The protein concentration of the supernatant corresponding to the crude lysate was measured by Bradford assay and adjusted to 4 mg/ml with lysis buffer; imidazole was added to a final concentration of 20 mM. For the pull-down assay, 100 µl of bead solution corresponding to 5 µl of magnetic beads (SERVA Ni-NTA Magnetic Beads) were used for each probe according to the manufacturer's protocol. Raw lysates and eluates from RGS6xHis-tagged PR72 and control-transfected HEK293 cells were loaded on a SDS polyacrylamide gel at a 5 to 1 ratio.

### Subcellular fractionation

Subcellular fractions were prepared from whole heart homogenates by differential centrifugation as described (18). This protocol allows the preparation of cardiomyocyte-derived fractions enriched in different cellular organelles. The supernatant obtained after the first centrifugation of heart homogenates represents the cytosol. It can be suggested that most of the fibroblasts, constituting the main fraction of non-cardiomyocyte cells, are enriched in the pellet after the first centrifugation. Thus, the cytosol fraction should contain mostly proteins derived from cardiomyocytes. Moreover, this preparation protocol enables the preparation of purified cardiac contractile myofibrils that are virtually free of contamination by mitochondrial, sarcolemmal, and sarcoplasmic reticulum (SR) membranes (19). The membrane-enriched fraction contains sarcolemmal as well as SR proteins. However, it cannot be excluded that fractions contain small quantities of non-cardiomyocyte cells.

### Histological examination

As described previously (20), TG and WT hearts were immersed into 4% saline-buffered formaldehyde and embedded in paraffin. Transversal sections at a thickness of 5 μm were mounted on glass slides. For estimation of interstitial fibrosis, sections were stained with Masson–Goldner's Trichrome. To estimate cell diameters, tissue sections were subjected to hematoxylin-eosin (HE) staining. Within each HE-stained section, 100 longitudinally oriented cells were randomly selected. The transverse diameters, which were orthogonal to the longitudinal cell axes, were subsequently quantified at the widest region of each selected cell. This measurement was performed using the two-point measurement tool available in the NIS-Elements AR software (Nikon Instruments).

### Protein phosphatase assay

The preparation of [^32^P]-labeled phosphorylase *α* is based on an established protocol (21). For the assay, heart samples obtained from Langendorff experiments (section “Heart tissue samples”) were mixed with a buffer that contained EDTA resulting in complexation of free Ca^2+^ and Mg^2+^ ions, which completely inhibits the catalytic activity of PP's, except for PP1 and PP2A. The dilution of the samples was chosen to convert no more than 18% of the maximum releasable [^32^P]. The dephosphorylation reaction was performed for 20 min at 30°C by adding 20 μl of phosphorylase α. Subsequently, an equal volume of 50% trichloroacetic acid was added to the mixtures which stopped the reaction. Moreover, 20 μl of ultrapure water and 30 μl of bovine serum albumin solution were added. The mixtures were centrifuged at 14,000 × g and 4°C for an additional 10 min after incubation on ice for 10 min. Thereafter, 50 μl of the supernatant was analyzed in the scintillation counter. The specific activity of the phosphorylase α substrate was calculated from the difference between total counts and background radioactivity. To distinguish between PP1 and PP2A, samples were incubated in the absence and presence of 3 nM okadaic acid. At this concentration, okadaic acid completely inhibits PP2A activity, leaving PP1 activity unaffected (22).

### Detection of cAMP levels

The cAMP-concentration of heart samples obtained from Langendorff experiments (section “Heart tissue samples”) were measured by Gs Dynamic HTRF Kit (CISBIO). According to manufacturer instructions, 10 µg protein for each sample were used. After 1 h of incubation fluorescence signals were detected with Mithras LB 940 Microplate Analyzer (Berthold Technologies, Bad Wildbad, Germany). cAMP-concentrations were calculated by a cAMP-standard curve in Origin 2022b software.

### Hemodynamic measurements

Hemodynamic measurements were performed according to an approved protocol (23). Experimental animals were anesthetized with an i.p. dose of 400 mg/kg of a 2% solution of tribromoethanol. For data acquisition, a 1.4 Fr pressure-volume catheter (model SPR-839) was inserted into the left ventricle. The catheter was optimally placed when sinusoidal pressure curves were recorded using the MPVS-400 system and LabChart software (ADInstruments). After a 10 min stabilization period, heart rates and left ventricular pressures were recorded and analyzed. At the end of the experiment, animals were killed by a repeated dose of tribromoethanol, followed by cervical dislocation in deep anesthesia.

### Sarcomere length shortening and Ca^2+^ transients

Contractile function and intracellular Ca^2+^ cycling of cardiomyocytes were studied by use of a dual emission photometry system combined with a CCD camera (Myocyte Calcium and Contractility Recording System; IonOptix Ltd.) attached to an epifluorescence microscope (Nikon). Cardiomyocytes were initially incubated with 23.3 μM Indo-1/AM (Molecular Probes) for 10 min in a small perfusion chamber mounted on the microscope. This chamber was perfused with bath solution (composition in mM: 140 NaCl, 5.8 KCl, 0.5 KH_2_PO_4_, 0.4 Na_2_HPO_4_, 0.9 MgSO_4_, 10 HEPES, 1 glucose, and 2 CaCl_2_, pH = 7.45), and cardiomyocytes were field-stimulated with 0.5 Hz under basal conditions as well as in the presence of 1 μM isoprenaline or 3 nM okadaic acid. The emitted fluorescence was recorded at wavelengths of 405 and 495 nm. The ratio of both wavelengths was taken as an index of cytosolic Ca^2+^ concentration. The maximum peak amplitude of Ca^2+^ transients was related to baseline (Δ Indo-1 ratio). The shortening of sarcomere length (SL) was detected simultaneously. The maximum SL shortening was normalized to resting sarcomere length (Δ SL shortening).

### Measurement of LTCC currents

Whole-cell patch-clamp recordings were performed in voltage clamp configuration to record voltage gated L-type Ca^2+^ currents (I_CaL_) in isolated ventricular myocytes. Measurements were conducted using the ruptured patch method and were performed at room temperature (22 ± 1°C, RT). Borosilicate glass capillaries (GB150TF-8P, Science Products, Hofheim, Germany) were pulled to a resistance of 3.5 ± 1 M*Ω*. Data was acquired and filtered at 10 kHz using an EPC-800 amplifier and sampled with an 18bit A/D converter InstruTech ITC-18 under the control of the PatchMaster software (HEKA Elektronik, Lambrecht, Germany). Series resistance was compensated for at least 60%. The bath solution contained (in mM): 136 NaCl, 5.4 KCl, 1.8 CaCl_2_, 1 MgCl_2_, 5 HEPES, 0.33 NaH_2_PO_4_, 10 TEA-Cl, 0.1 BaCl_2_, and 10 glucose, pH 7.4. Pipettes were filled with a solution containing (in mM): 120 CsCl, 1 MgCl_2_, 5 Na_2_ATP, 10 TEA-Cl, 10 EGTA, and 10 HEPES, pH 7.2. Current density (pA/pF), current-voltage (IV) relations and voltage dependence of activation and inactivation were calculated as described before (24, 25).

### SR Ca^2+^ load

Measurement of SR Ca^2+^ release was performed according to a revised protocol (26). Briefly, isolated cardiomyocytes were perfused with Tyrode's solution and at least 10 Ca^2+^ transients were recorded at 0.5 Hz using the IonOptix system as described for the detection of Ca^2+^ transients. After a 10 s pause in stimulation, cells were superfused with 10 mM caffeine for 45 s using a rapid application aid. Within a few seconds, the cardiomyocytes responded immediately with a complete SR Ca^2+^ release and increased contraction (27). Data were recorded and analyzed, whereby the SR Ca^2+^ content corresponds to the maximum (peak) amplitude of the caffeine-induced Ca^2+^ transient.

### Ca^2+^ spark measurements

Cardiomyocytes were incubated with 4 μM Fluo-4 in the perfusion chamber of a confocal microscope (LSM710; Zeiss). Excess dye was removed, and cells were then perfused with Tyrode's solution. During a pause in stimulation, a line scan (X-T scan) was performed (excitation: 488 nm; emission range: 505–622 nm). Detection and analysis of Ca^2+^ sparks were performed using ImageJ software (https://imagej.nih.gov/ij/) and the Sparkmaster plugin (28).

### Heart tissue samples

Samples of cardiac tissues were collected from TG and WT mice. The mice were either left untreated or treated with an intraperitoneal injection of 4 mg isoprenaline per kg body weight. Two minutes after injection, the hearts were removed from euthanized mice, washed, and rapidly frozen in liquid nitrogen. Additional samples were obtained from spontaneously beating TG and WT hearts, retrogradely perfused in a Langendorff apparatus. After a stabilization period of 25 min, the hearts were either left untreated or stimulated with 1 µM isoprenaline for 10 min. The beating hearts were promptly frozen in liquid nitrogen.

### RT-PCR

Quantitative real-time PCR of WT and TG heart tissue samples was performed as previously described with minor modifications (20). Addionally, mRNA levels of Gapdh (Glyceraldehyde 3-phosphate dehydrogenase) were determind using AAC TTT GGC ATT GTG GAA GG as a forward and GGA TGC AGG GAT GAT GTT CT as a reverse primer sequence.

### SDS-PAGE and immunoblotting

Heart homogenates were prepared following the previously described method (29). For immunoblot analysis, the heart homogenates were mixed with reducing Laemmli loading buffer to achieve a final SDS concentration of 2.5%. The samples were then incubated at 30°C for 10 min and separated on either 10% SDS polyacrylamide gels or 4%–15% Criterion TGX Precast gels (BioRad). The proteins were transferred from the gels to nitrocellulose membranes using an electric current of 3 or 6 Ah. The membranes were incubated overnight at 4°C with specific antibodies against the following proteins: PLN (1:2000, Badrilla), PLN phospho-Ser^16^ (1:1000, Badrilla), PLN phospho-Thr^17^ (1:1000, Badrilla), RyR (1:1000, Thermo Fisher Scientific), RyR phospho-Ser^2808^ (1:1000, Badrilla), RyR phospho-Ser^2814^ (1:5000, Badrilla), TNI (1:1000, Cell Signaling), TNI phospho Ser^23/24^ (1:1000, Cell Signaling), MyBP-C (1:1000, Proteintech Group), MyBP-C phospho Ser^282^ (1:1000, Enzo), PKA-C*α* (1:1000, Cell Signaling), CaMKII (1:1000, Thermo Fisher Scientific), Cav1.2 (1:500, Alomone), SERCA2a [1:1000, (30)], NCX (1:1000, Swant Inc.), PPP2R3A/PR72 (1:1000, Sigma Aldrich), PPP2CA (1:2000, Proteintech Group), CSQ2 (1:2500, Thermo Fisher Scientific). The bound antibodies were detected by incubating the membranes with secondary antibodies (ECL rabbit/mouse IgG, HRP-linked whole antibody, GE Healthcare) for 2 h. The signals were visualized using the SERVALight Helios CL HRP WB Substrate kit (Serva) and a ChemicDoc XRS detection system. The antibody signals were quantified using the TotalLab TL120 software.

### Data analysis

The results are presented both in terms of individual data points and as means with corresponding standard deviations (+SD), if not indicated otherwise. In the context of single-cell measurements, the data points represent individual cardiomyocytes (“*n*”), whereas in the context of multicellular measurements, the data points correspond to individual hearts or mice (“*N*”). Statistical analyses were conducted using either GraphPad Prism (Graphpad Software Inc.) or SigmaPlot software (Systat Software GmbH). To assess the normality of residuals, the following tests were employed: Anderson-Darling (A2*), D'Agostino-Pearson omnibus (K2), Shapiro-Wilk (W), and Kolmogorov-Smirnov (distance) tests. Depending on the specific research question, the comparison groups being examined, and the normality characteristics of the residuals, various statistical tests were utilized. The respective tests are indicated in the figure captions. Statistical significance was determined by a threshold of *P *< 0.05, denoting group differences that were considered to be statistically significant.

## Results

### Functional interaction between PR72 and PP2Ac

**Figure 1:**
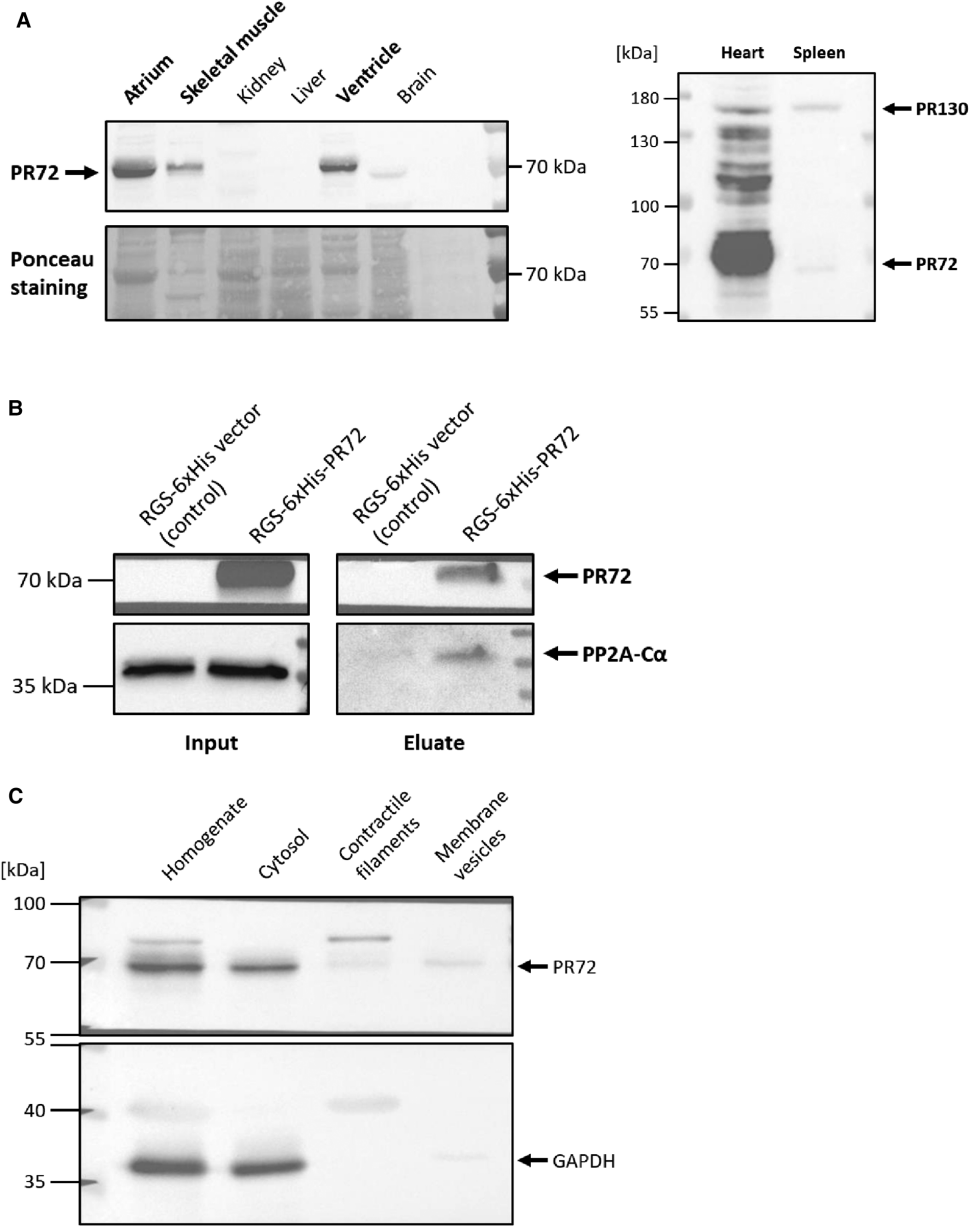
Direct interaction between PR72 and PP2Ac. The expression of PR72 in different tissues is shown in the left panel (**A**) In the murine heart, PR72 is the more abundant *PPP2R3A* splice variant when compared to PR130, as shown in the right panel (**A**) The physical interaction between PR72 and PP2Ac was also demonstrated by pulldown of the catalytic subunit on immobilized 6xHis-PR72 (**B**) The recombinant expression of the 6xHis-tagged PR72 (lane 2) was performed in HEK293 cells and controlled using an RGS-6xHis control vector (lane 1). Note the expression of the endogenous PP2Ac in the cells (left panel). Elution of PR72 from the Ni-NTA column using imidazole identified PP2Ac as a binding partner of the regulatory B'' subunit (right panel). Subcellular fractions were prepared from pulverized heart tissue of WT mice by differential centrifugation (**C**) PR72 was found to be mainly expressed in the cytosol. The purity of the cytosolic fraction was tested by using a specific antibody directed against GAPDH.

First of all, we examined the expression level of PR72 in different tissues. We found that PR72 is mainly expressed in the myocardium, including the ventricles and atria, and represents the more abundant *PPP2R3A* splice variant in the mouse heart (Figure 1A). After clarifying the expression of PR72, the question arose as to whether this regulatory B'' subunit interacts with other subunits of the PP2A holoenzyme. Biochemical experiments revealed a direct interaction between PR72 regulatory and catalytic subunits by immobilizing 6xHis-tagged PR72 on Ni columns, which resulted in pulldown of PP2Ac (Figure 1B). Recombinant PR72 was expressed in HEK293, and the total cell lysate for the assay was also prepared. After investigating the organ-specific expression of PR72 and its interaction with PP2Ac, we examined the intracellular localization of PR72 in cardiac tissue. PR72 was mostly detected in the cytosolic fraction of the dissected heart tissue (Figure 1C). The purity of the cytosolic fraction was demonstrated using two different marker proteins (GAPDH and myoglobin) in the immunoblot analyses (Figure 1C, Supplementary Figure 1). The enrichment of membrane preparation and contractile filaments were also examined using specific marker proteins (Supplementary Figure 1).

### Cardiac-specific overexpression of PR72 is associated with unchanged PP2A activity

**Figure 2:**
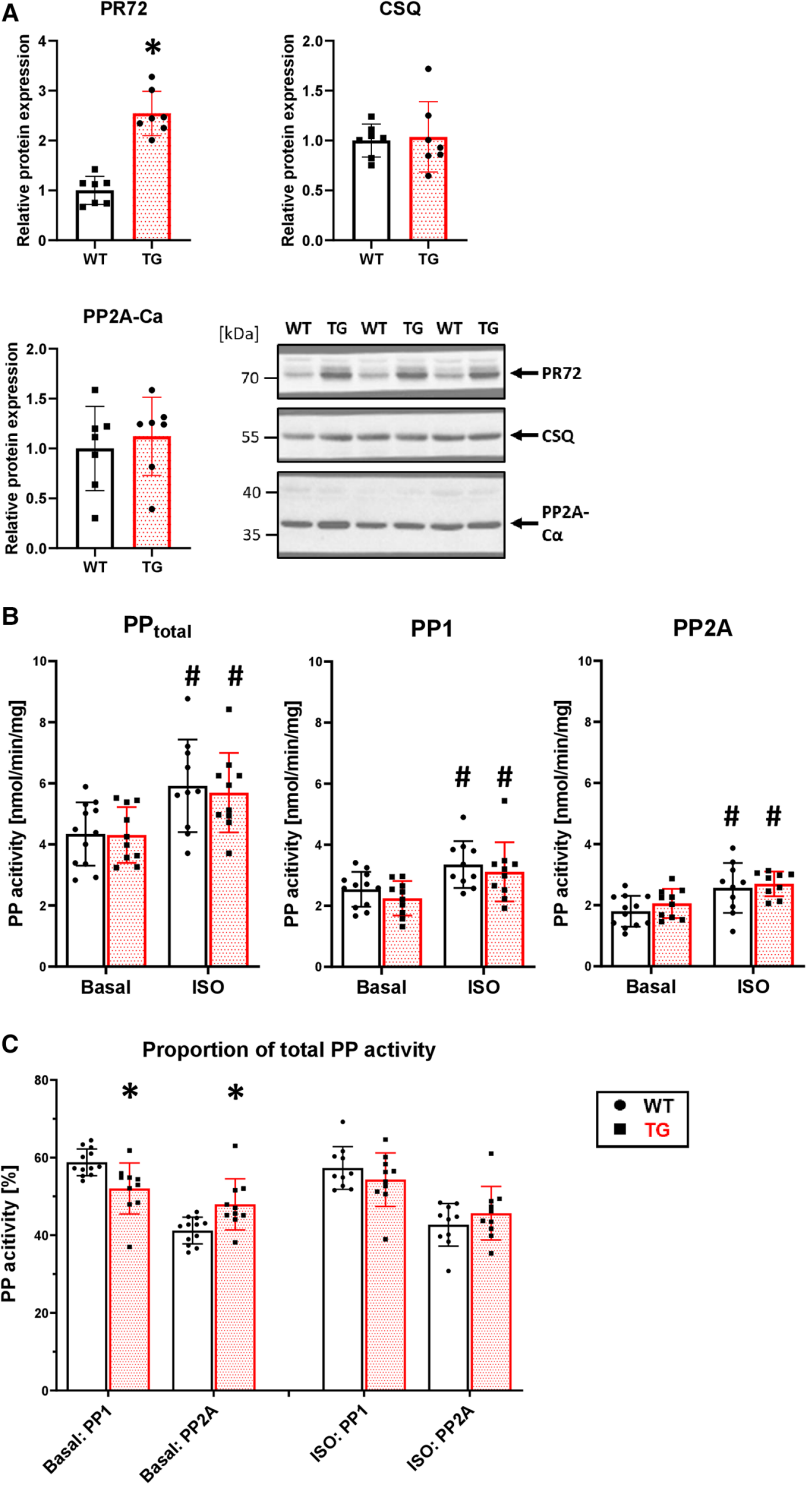
2.5-fold overexpression of PR72 in TG hearts. Overexpression of PR72 was confirmed using a specific antibody for immunoblotting (**A**) Additionally, the protein expression of the catalytic subunit of PP2A (Ca) and calsequestrin (CSQ) was determined (**A**), with representative immunoblots shown for heart homogenates of transgenic (TG) and wild-type (WT) mice (*P < 0.05 vs. WT, *N* = 7 hearts each, unpaired Student's *t*-test). Proteins were normalized to total protein content measured in Ponceau stainings. Protein phosphatase activities, including total, PP1, and PP2A, were measured in homogenates of Langendorff-perfused hearts under basal conditions and after *β*-adrenergic stimulation with isoprenaline (**B**) (^#^*P *< 0.05 vs. Basal, *N* = 10-12 hearts, 2-way-ANOVA and Holm–Sidak post-hoc test). The proportion of PP2A on total PP activity is depicted (**C**) (*P < 0.05 vs. WT, *N* = 10-12 hearts, Kruskal–Wallis One-way-ANOVA on Ranks and Dunn's post-hoc test).

To test the functional relevance of PR72, we generated a heart-directed transgenic mouse model under the control of the well-established *α*MHC promoter. Southern blot analysis identified a total of six founders (Supplementary Figure 2A), with one dying and another producing only wild-type descendants. Founder line 4 exhibited the highest level of overexpression (2.5-fold) compared to wild-type (WT) littermates (Figure 2A). This line was used for further characterization of the transgenic overexpression model because the expression level of PR72 is comparable to that measured in human heart failure (12). Of the remaining three founders, only line 3 showed weak overexpression (Supplementary Figure 2B). Overexpression of PR72 was accompanied by unchanged expression of PP2Ac and calsequestrin, a myocardial marker protein (Figure 2A). Under basal conditions, there was no difference in PP2A activity between TG and WT littermates (Figure 2B). Stimulation of Langendorff-perfused hearts with 1 μM isoprenaline increased PP2A activity, but there was no difference between genotypes (Figure 2B). Interestingly, the ratio of PP2A to total PP activity changed between genotypes after isoprenaline application (Figure 2C); in other words, the initially increased proportion of PP2A normalizes after *β*-adrenergic stimulation.

**Figure 3:**
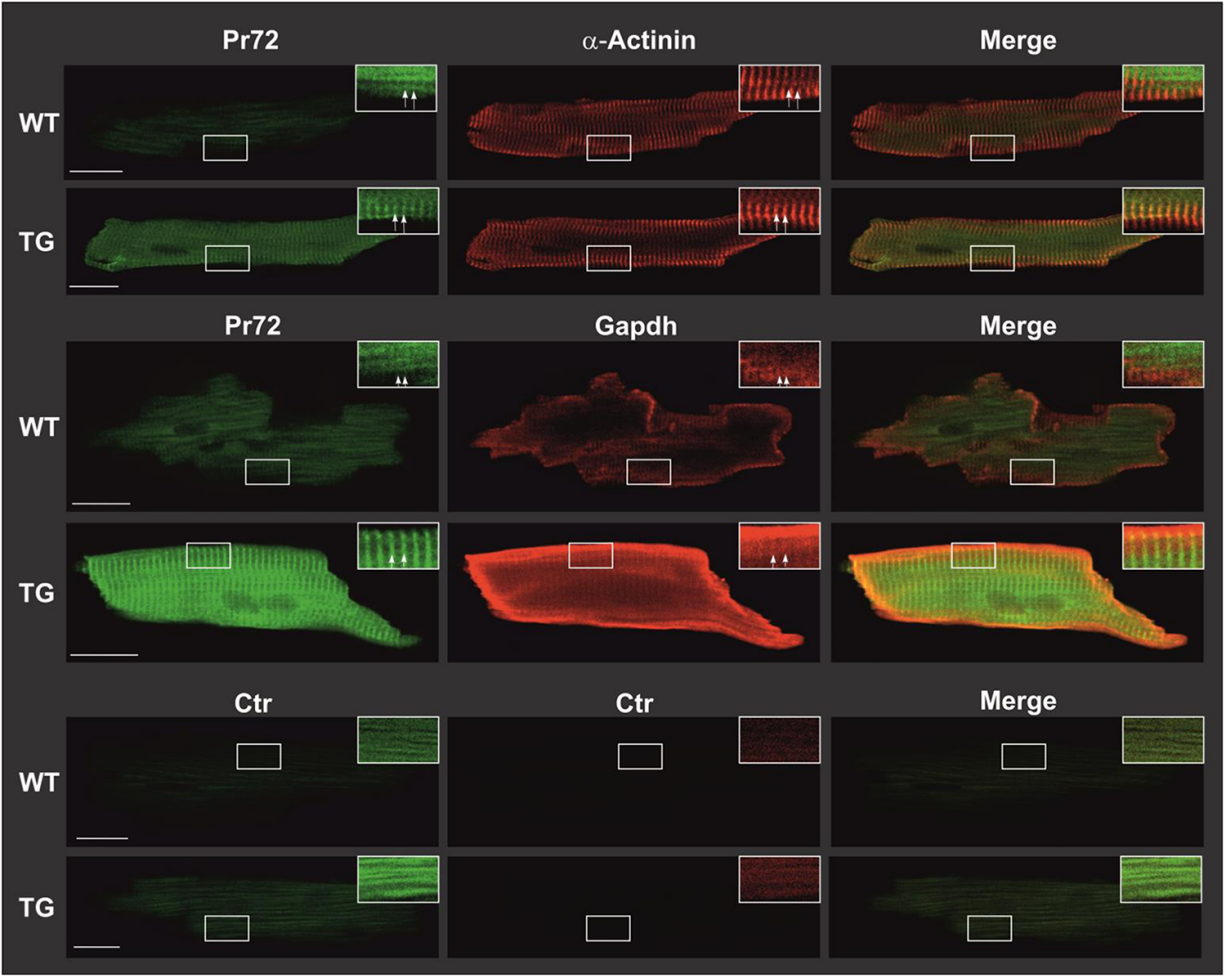
Intracellular distribution of PR72 and marker proteins in TG and WT cardiomyocytes. Cardiomyocytes were isolated from TG and WT mice. Subsequently, they were fixed with formaldehyde on polylysine-coated glass slides and permeabilized using tergitol. Nonspecific binding was minimized by employing BSA and goat serum as blocking agents. Immunostaining procedures were conducted on the cardiomyocytes using anti-PR72 antibody in conjunction with either anti-sarcomeric *α*-actinin antibody or anti-GAPDH antibody. The primary antibodies were then detected using fluorescence-coupled secondary antibodies and analyzed through confocal laser microscopy. The expression of PR72 was visualized in green, while other marker proteins were visualized in red, resulting in a yellow coloration where they colocalized. White scale bars represented a 20-µm reference. For improved clarity, specific regions of interest were magnified, and adjustments to contrast and brightness were made (indicated by white framed boxes). Striated regions were exemplarily marked with white arrows. In the lower panel, negative controls demonstrated cardiomyocyte autofluorescence. In all staining experiments, 10 cells per heart from each genotype were examined, and representative results are shown.

Overexpression was confirmed in immunocytotological measurements (Figure 3). In WT cardiomyocytes, the expression of PR72 was only minimally higher than in negative controls. In a study conducted by DeGrande et al. (12), expression of PR72 along the Z line was detected for the first time in cardiomyocytes. Our study also used confocal microscopy and revealed a striated pattern, that was more pronounced in TG, indicating a sarcomeric organization of PR72 at the Z line (Figure 3). Detailed analysis of PR72 and sarcomeric *α*-actinin localization showed a match of both proteins (see white arrows), suggesting direct colocalization. A similar pattern was also demonstrated for the detection of GAPDH (a marker of the cytosol) and PR72 (Figure 3). Previous work showed no staining of any specific structure by PR72 (31) in cross-sections of cardiac muscle fibers, suggesting a cytosolic localization. In our immunocytological studies in cardiomyocytes, we detected a diffuse distribution of PR72 in addition to the striated pattern (Figure 3). This may hind to a cytosolic localization of this regulatory subunit of PP2A. In contrast, the negative control showed only autofluorescence.

### Mild heart hypertrophy in PR72-overexpressing mice

**Figure 4:**
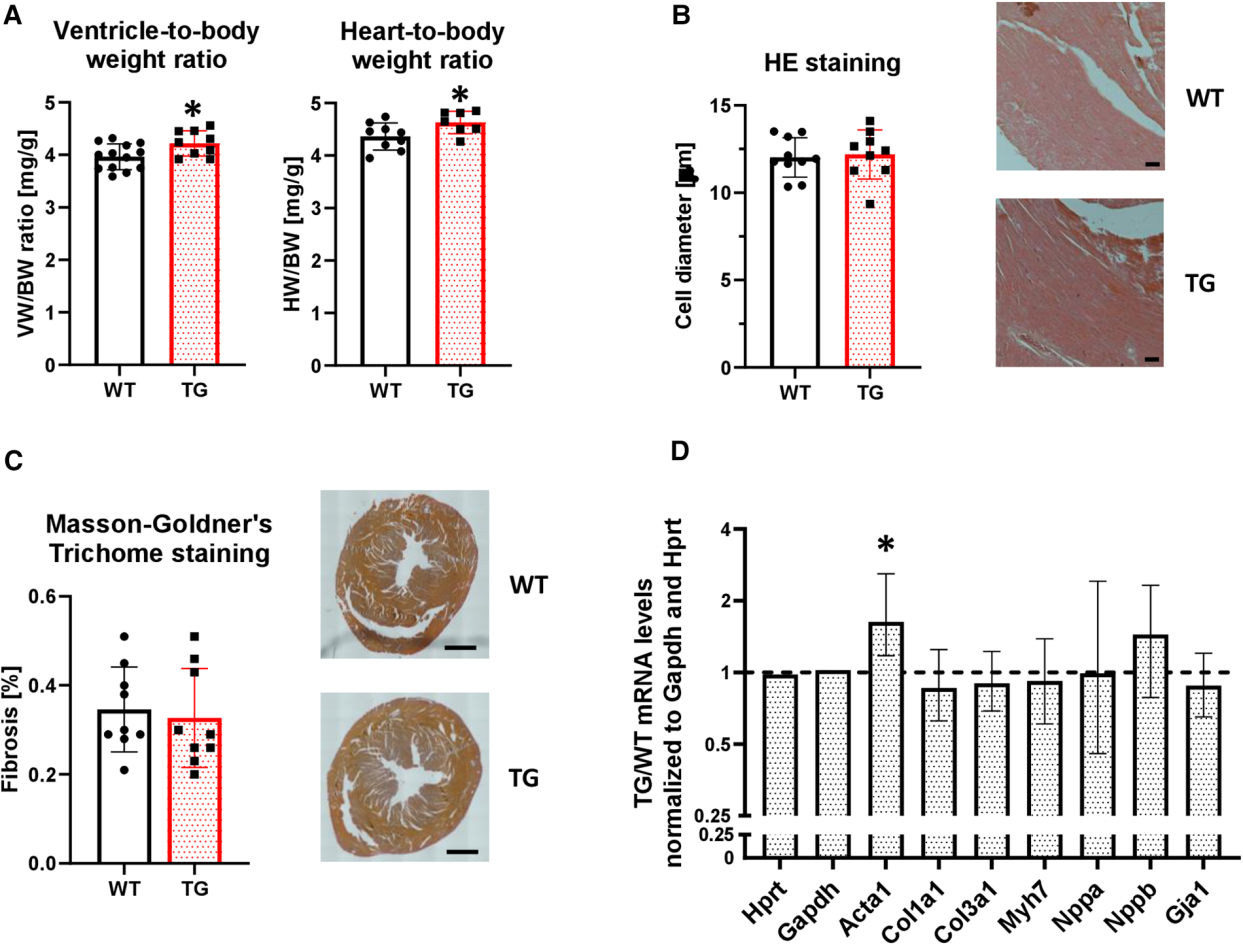
Overexpression of PR72 results in moderate cardiac hypertrophy. To assess the development of cardiac hypertrophy in transgenic mice, the heart-body weight (HW/BW, *N* = 7-10 hearts) and ventricle-body weight (VW/BW, *N* = 9-13 hearts) ratios were determined (**A**) (*P < 0.05 vs. WT, unpaired Student's *t*-test). The cardiomyocyte diameter was determined on hematoxylin and eosin stained tissue sections (**B**) Exemplary sections for both genotypes are shown on the right hand side (*N* = 9-10 hearts). The black scale bars show a 50 µm distance. The degree of fibrosis was analyzed in ventricular sections stained with Masson-Goldner's Trichrome (**C**) (*N* = 9 hearts each). Black scale bars indicate a 1 mm distance. mRNA expression levels were determined for selected markers of a hypertrophic gene program (Acta1, skeletal muscle *α*-actin; Col1a1, collagen type I *α*_1_ chain; Col3a1, collagen type III *α*_1_ chain; Gja1, connexin 43; Myh7, myosin heavy chain beta (MHC-*β*) isoform; Nppa, atrial natriuretic peptide; Nppb, brain natriuretic peptide) (**D**) The mRNA expression levels were normalized to the reference genes Hypoxanthine-guanine phosphoribosyltransferase (Hprt) and Gapdh. RT-PCR data were analyzed using the relative expression software tool (REST© Version 2.013) and shown as relative mRNA levels ± standard error (*P < 0.05 vs. WT, *N* = 4-7 hearts).

Overexpression of PR72 was associated with a mild cardiac hypertrophy (Figure 4A), which was not sex-specific. This was not associated with comparable changes at the level of single cardiomyocytes (Figure 4B). Signs of cardiac decompensation could be excluded because both lung and liver weights were unchanged (Supplementary Figure 2C), and there were no signs of developing fibrosis (Figure 4C). However, mRNA levels of skeletal muscle *α*-actin (*ACTA1*), which marks the earliest appearance in embryonic cardiomyocytes and, therefore, represents a marker of fibrogenic cell activity, were enhanced in TG compered to WT hearts (Figure 4D). Other marker proteins of hypertrophy and fibrosis tested were unchanged between genotypes.

### Enhanced contractility in intact transgenic mice and isolated cardiomyocytes

**Figure 5:**
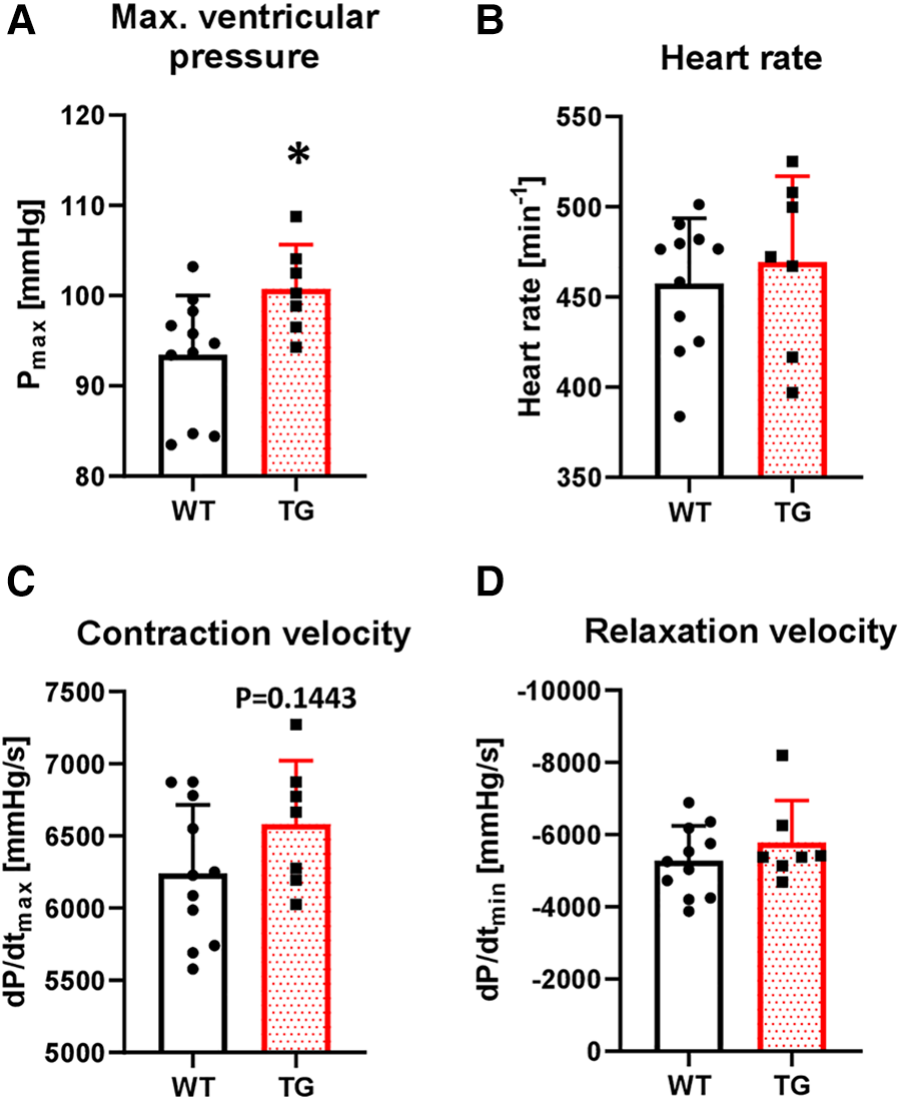
Increased left ventricular pressure in TG mice. The basal contractile parameters of catheterized transgenic (TG) and wild-type (WT) mice were monitored. The summarized data show the maximal ventricular pressure (**A**), heart rate (**B**), contraction velocity (**C**), and relaxation velocity (**D**) (*P < 0.05 vs. WT, *N* = 7-11 mice, unpaired Student's *t*-test).

To study the functional effects of PR72 overexpression on contractility, we first conducted *in vivo* left ventricular catheterization. We found that basal maximal ventricular pressure (P_max_) increased by 8% in TG animals compared to WT (Figure 5A). There were no chronotropic effects on this increase (Figure 5B). The contraction and relaxation velocities (dP/dt_max_ and dP/dt_min_) showed a tendency towards an increase compared to WT (Figures 5C,D).

**Figure 6:**
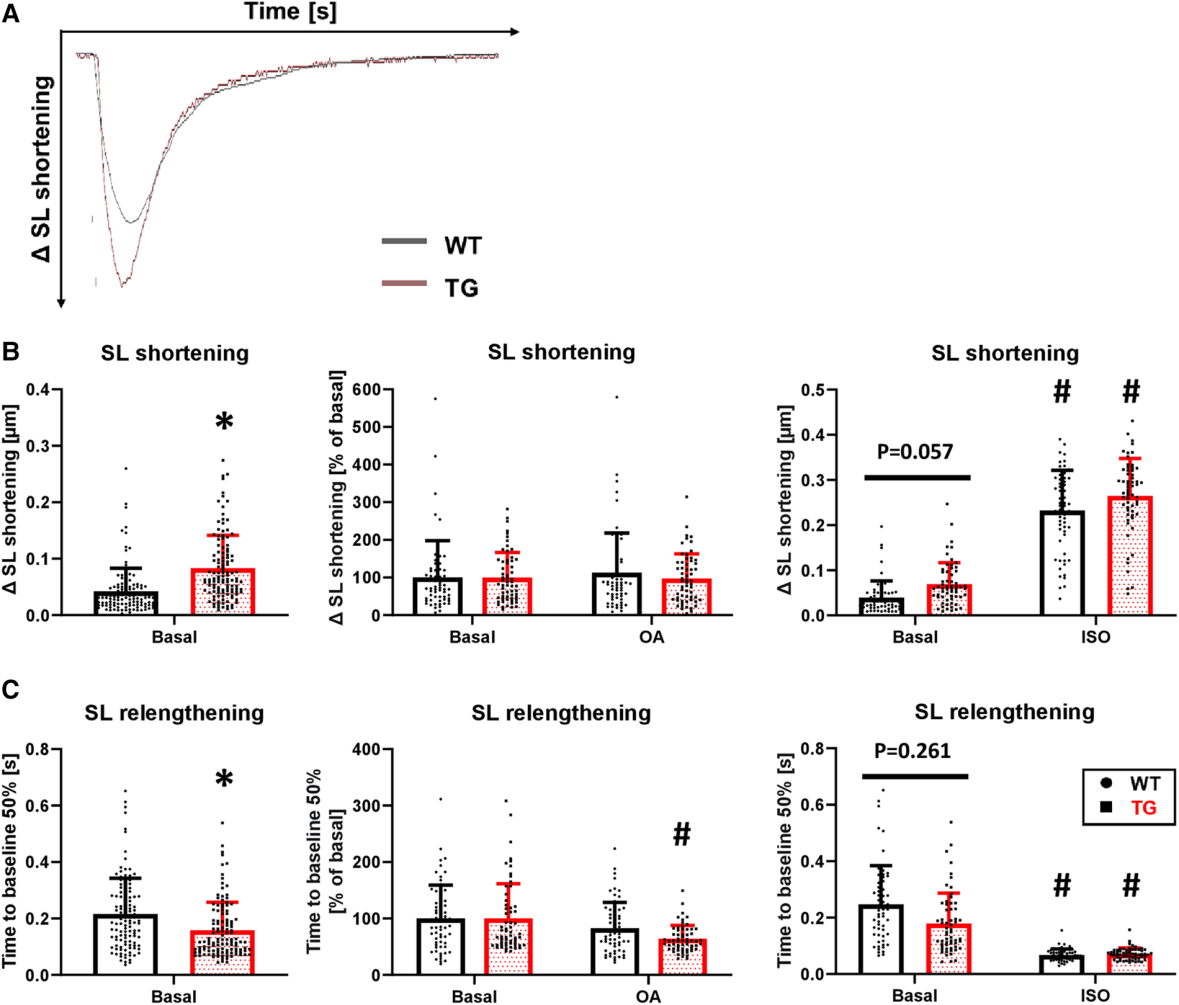
Enhanced contraction and hastened relaxation in TG cardiomyocytes. Myocellular contraction parameters were determined in electrically stimulated (0.5 Hz) cardiomyocytes of both WT and TG mice. Representative tracings of myocellular contractions are shown (**A**), and summarized data of maximal sarcomere length (SL) shortening under basal conditions, in the presence of 3 nM okadaic acid (OA), or after stimulation with 1 μM isoprenaline (ISO) are presented (**B**) Additionally, the time to 50% relengthening of cardiomyocytes is shown for each of the study conditions described above (**C**) (basal conditions (left panels): *n*/*N* = 120 cardiomyocytes/12 hearts, *P < 0.05 vs. WT, Mann–Whitney test; ISO (right panels) and OA (central panels): *n*/*N* = 60 cardiomyocytes/6 hearts, respectively, *P < 0.05 vs. WT, #*P *< 0.05 vs. Basal, Kruskal–Wallis One-way-ANOVA on Ranks and Dunn's post-hoc test).

To determine whether the increased left ventricular pressure measured in the whole animal by catheterization also occurs at the single-cell level, we isolated cardiomyocytes by enzymatic digestion and electrically stimulated them at 0.5 Hz in a perfusion chamber (Figure 6A). Under basal conditions, sarcomere length (SL) shortening was increased by 97% in TG cardiomyocytes compared to WT (Figure 6B). Application of 3 nM okadaic acid had no further effect on either genotype (Figure 6B). Under *β*-adrenergic stimulation, the increase in SL shortening observed under basal conditions in TG cardiomyocytes was abolished (Figure 6B). The relaxation of transgenic cardiomyocytes, measured as the time to 50% SL relengthening, was 26% faster compared to WT (Figure 6C). The improved relaxation in TG seemed to be influenced by PP2A because the administration of 3 nM okadaic acid further shortened this parameter (Figure 6C). The administration of 1 *μ*M isoprenaline resulted in an unchanged SL relengthening between TG and WT cardiomyocytes (Figure 6C).

### Increased Ca^2+^ transient peak amplitude but unchanged SR Ca^2+^ load and I_CaL_ in TG cardiomyocytes

**Figure 7:**
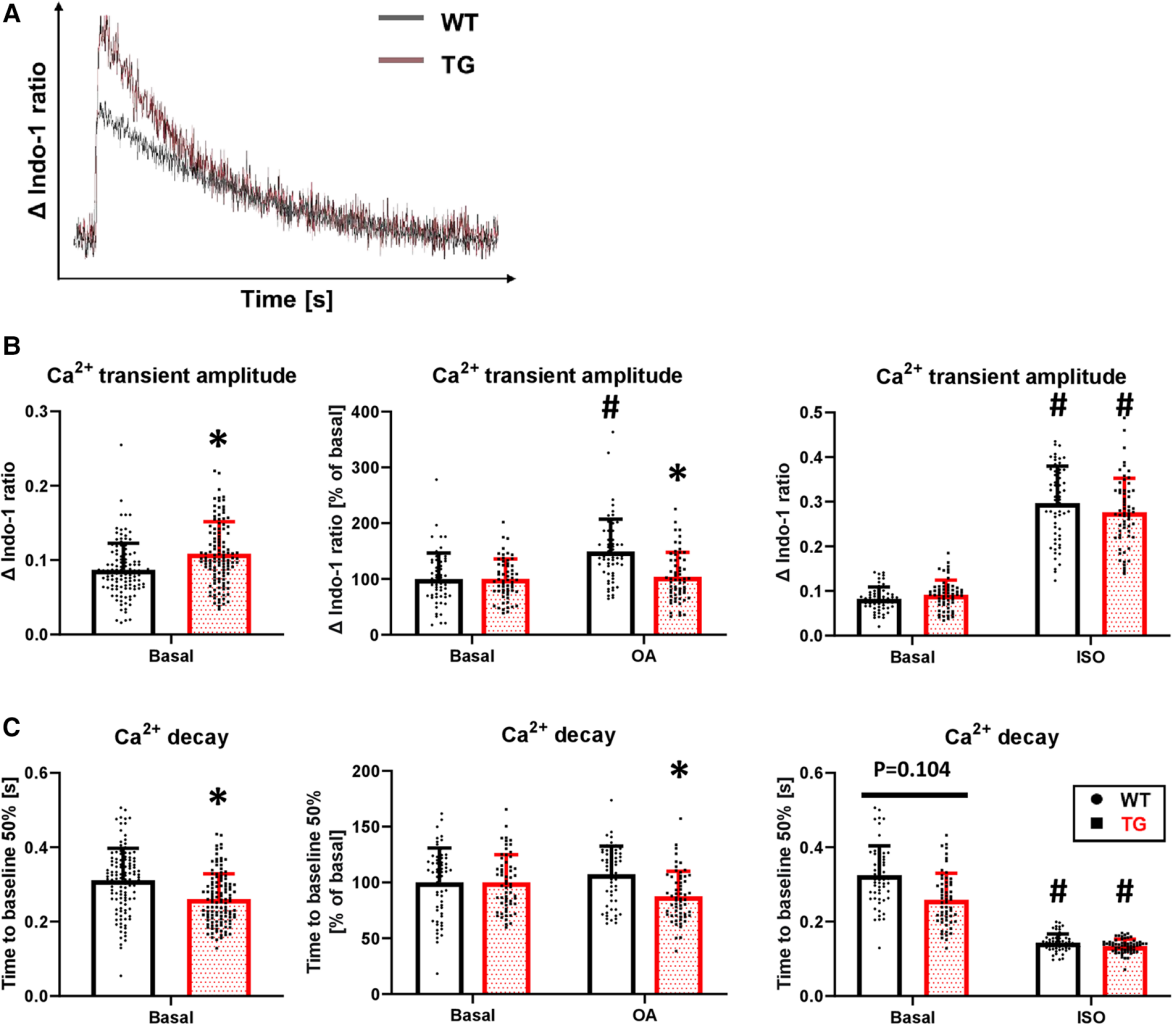
Increased peak amplitude and faster decay of Ca^2+^ transients in TG cardiomyocytes. Intracellular Ca^2+^ transients were determined in TG and WT cells by loading them with indo-1. Representative tracings of intracellular Ca^2+^ transients are shown (**A**) The peak amplitude of Ca^2+^ transient ratios was measured under basal conditions and after the application of okadaic acid (OA) or isoprenaline (ISO). Summarized data are provided (**B**), and summarized data of the time to 50% decay are presented for each of the study conditions described above (**C**) (basal conditions (left panels): *n*/*N* = 120 cardiomyocytes/12 hearts, *P < 0.05 vs. WT, Mann–Whitney test; ISO (right panels) and OA (central panels): *n*/*N* = 60 cardiomyocytes/6 hearts, respectively, *P < 0.05 vs. WT, #*P *< 0.05 vs. Basal, Kruskal–Wallis One-way-ANOVA on Ranks and Dunn's post-hoc test).

Concomitant with the increased SL shortening, there was an 25% increase in the peak amplitude of Ca^2+^ transients under basal conditions in TG cardiomyocytes compared to WT (Figures 7A,B). Inhibition of PP2A increased the peak amplitude of Ca^2+^ transients only in WT cardiomyocytes, while it remained unchanged in TG (Figure 7B). Administration of isoprenaline demonstrated an unchanged peak amplitude of Ca^2+^ transients between the two genotypes (Figure 7B), as observed for SL shortening (Figure 6B). The faster decay in the relaxation time in TG cardiomyocytes compared to WT (Figure 6C) was paralleled by corresponding effects for the Ca^2+^ transients (Figure 7C). This effect was sensitive to PP2A inhibition, as the administration of 3 nM okadaic acid resulted in an enhancement of the Ca^2+^ transient decay only in TG (Figure 7C). Isoprenaline augmented the Ca^2+^ transient decay in both genotypes to different extents compared to basal conditions but towards comparable final effects (Figure 7C).

**Figure 8:**
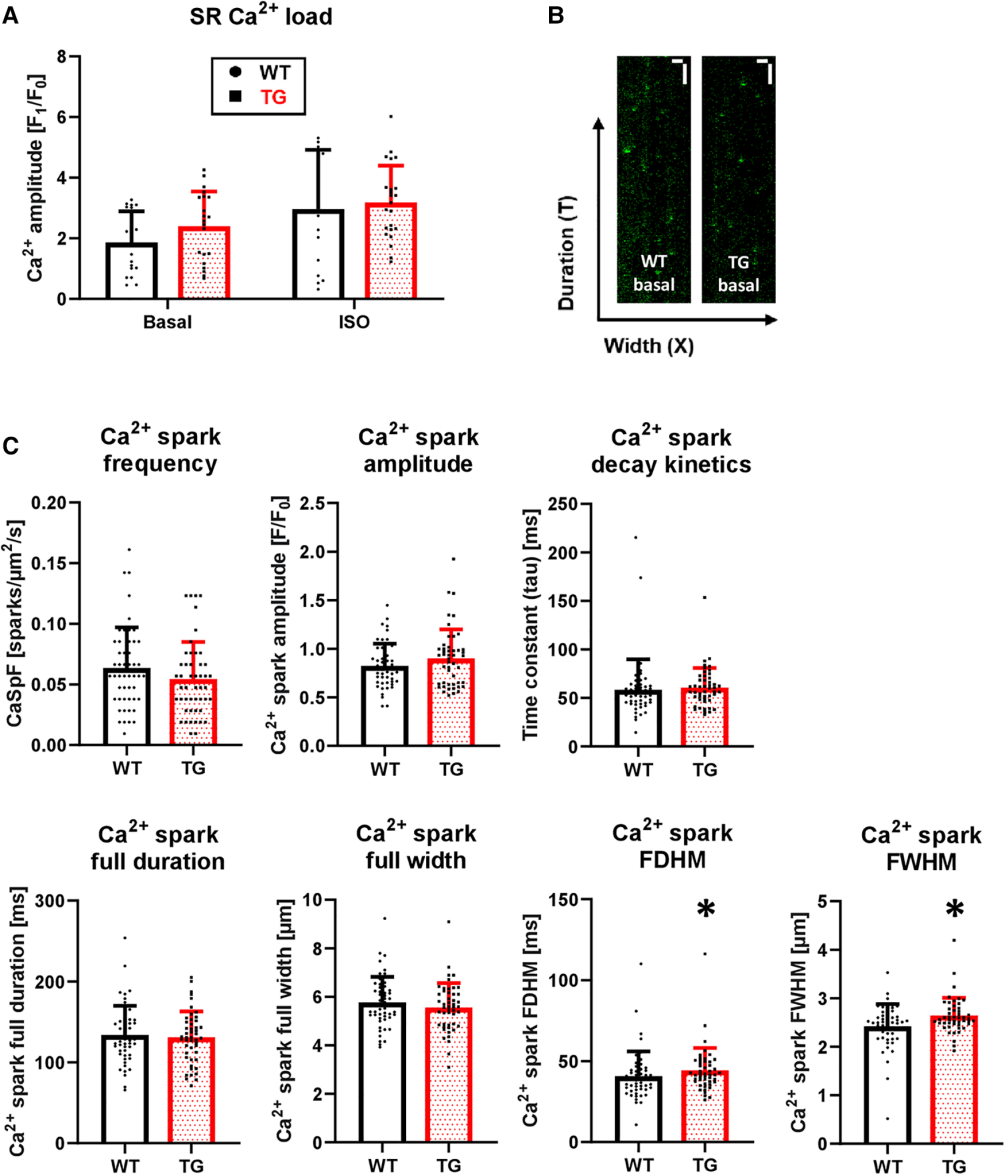
Unchanged SR Ca^2+^ load but enhanced Ca^2+^ spark parameters in TG cardiomyocytes. Caffeine-induced Ca^2+^ transients were measured in isolated myocytes to estimate the SR Ca^2+^ load. The quantitative analysis of peak amplitude is shown for TG and WT in the absence and presence of 1 μM isoprenaline (**A**) (*n*/*N* = 15-23 cardiomyocytes/3 hearts, Kruskal–Wallis One-way-ANOVA on Ranks and Dunn's post-hoc test). Additionally, Ca^2+^ spark parameters were determined using laser scanning microscopy. Characteristic X-T scans of WT and TG cardiomyocytes after 1 Hz prestimulation are illustrated (**B**) The white scale bars indicate horizontally a 10-µm width and vertically a 1-sec duration. The quantified data for Ca^2+^ spark frequency (CaSpF), amplitude, full duration and full width, full duration and width at half maximum (FDHM and FWHM, respectively), and decay kinetics are shown (**C**) (*P < 0.05 vs. WT, *n*/*N* = 48-50 cardiomyocytes/5 hearts, Mann–Whitney test).

The increased amplitude and hastened decay of Ca^2+^ transients were accompanied by an unchanged SR Ca^2+^ load under both basal conditions and *β*-adrenergic stimulation (Figure 8A). To test whether an altered trigger was responsible for the increased Ca^2+^ transient amplitude in TG, we determined the Ca^2+^-induced Ca^2+^ release gain by measuring *I*_CaL_. However, the peak current density was comparable between TG and WT (−10.2 ± 2.7 vs. −10.0 ± 2.8 pA/pF, respectively, *n*/*N* = 26–27 cardiomyocytes/5 hearts, unpaired Student's *t*-test). In addition, the *I*_CaL_ inactivation kinetics were unchanged in TG compared to WT (*τ*_1_: 10.8 ± 7.7 vs. 10.6 ± 5.2 ms, respectively; *τ*_2_: 71.7 ± 8.6 vs. 72.7 ± 6.4 ms, respectively, *n*/*N* = 26–27 cardiomyocytes/5 hearts, Mann–Whitney *U*-test).

### Increased spatial spread of Ca^2+^ sparks in transgenic cardiomyocytes

To investigate the influence of potentially altered release characteristics of the junctional SR complex on Ca^2+^ transients, we determined various Ca^2+^ spark parameters (Figure 8B). The Ca^2+^ spark frequency, which most closely reflects the number of individual or clustered RyRs, was correspondingly unchanged between TG and WT cardiomyocytes (Figure 8C). Ca^2+^ spark amplitude tended to be increased in TG cardiomyocytes but did not reach significance (Figure 8C). The full duration and the full width of Ca^2+^ sparks were unchanged between both genotypes (Figure 8C). However, the spatial spread or size of Ca^2+^ sparks, measured as the full duration at half maximum (FDHM) and the width at half maximum (FWHM), were increased in TG compered to WT cardiomyocytes (Figure 8C). Lastly, the decay of Ca^2+^ sparks was unaffected between TG and WT cells (Figure 8C).

### Increased phosphorylation of phospholamban at threonine-17 and reduced NCX expression in transgenic hearts

**Figure 9:**
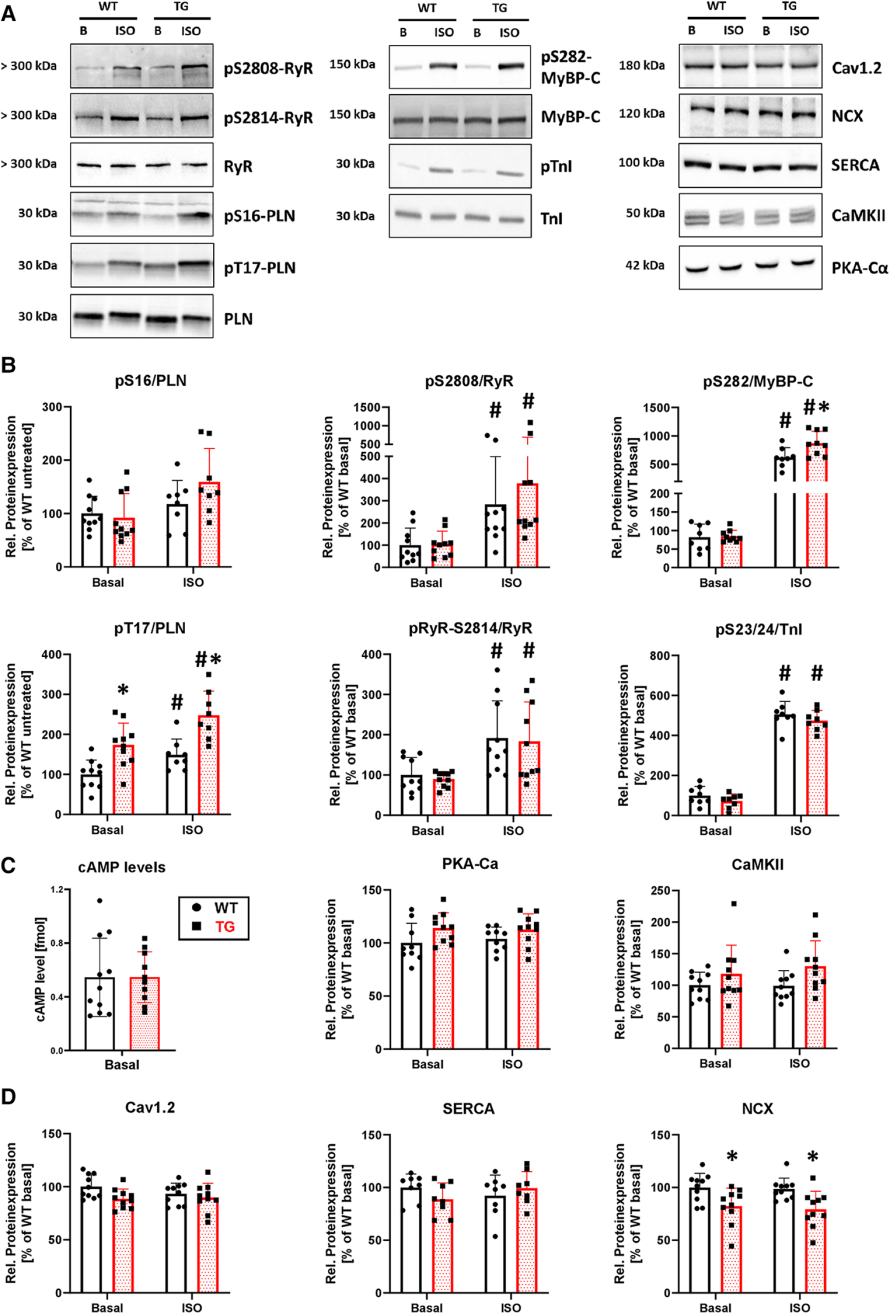
Higher phosphorylation of phospholamban at threonine-17 and lower expression of NCX in TG hearts. Shown are representative immunoblots of cardiac proteins involved in calcium regulation and contractility, for both TG and WT mice (**A**) Protein phosphorylation was measured in the presence and absence of isoprenaline (ISO). Molecular weight markers were included on the left-hand side of the blots. Protein phosphorylation for sarcoplasmic reticulum (PLN, phospholamban; RyR2, ryanodine receptor type 2) and myofilament proteins (MyBP-C, myosin-binding protein C; TnI, troponin inhibitor) was quantified under both basal and stimulated (ISO) conditions (**B**) Phosphorylation levels were normalized to the nonphosphorylated form of the individual protein and subsequently adjusted to WT levels under basal conditions (*P < 0.05 vs. WT, ^#^*P *< 0.05 vs. Basal, *N* = 8-10 hearts, 2-way-ANOVA and Holm–Sidak post-hoc test or, for pS2808/RyR, Kruskal–Wallis One-way-ANOVA on Ranks and Tukey post-hoc test). The *β*-adrenergic signaling was assessed by measurement of cAMP levels (*N* = 10-11 hearts) and the protein expression level of the catalytic subunit of cAMP-dependent protein kinase (PKA-C*α*, *N* = 9-10 hearts) (**C**) In addition, the protein expression of the Ca^2+^/calmodulin-dependent protein kinase II (CaMKII, *N* = 10 each) was measured in both genotypes (**D**) Expression levels of cardiac Ca^2+^ regulatory proteins [Cav1.2, L-type Ca^2+^ channel; NCX, Na^+^/Ca^2+^ exchanger; SERCA, sarcoendo(plasmic) reticulum Ca^2+^ ATPase] was analyzed under basal conditions and after stimulation with ISO in TG and WT (*P < 0.05 vs. WT, *N* = 8-10 hearts, 2-way-ANOVA and Holm–Sidak post-hoc test). Non-phosphorylated proteins were normalized to total protein content measured in Ponceau stainings.

To determine whether the observed contractile and intracellular Ca^2+^ cycling effects were accompanied by corresponding biochemical changes, we investigated the phosphorylation state of key proteins involved in contractility and Ca^2+^ regulation (Figure 9A). Our findings indicated that, under basal conditions, the phosphorylation of phospholamban at threonine-17 was increased by 74% in TG compared to WT (Figure 9B). However, phosphorylation of phospholamban at serine-16, ryanodine receptor type 2 at serine-2808 and serine-2814, myosin-binding protein C at serine-282, and troponin inhibitor at serine-23/24 remained unchanged (Figure 9B). Following *β*-adrenergic stimulation, the phosphorylation of both phospholamban at threonine-17 and myosin-binding protein C increased in TG compared to WT (Figure 9B). Notably, phosphorylation levels for the other proteins remained unchanged between genotypes under isoprenaline. To investigate whether changes in the *β*-adrenergic signaling are responsible for the phosphorylation effects, we measured cAMP levels and the protein expression of the catalytic subunit of cAMP-dependent protein kinase. Both parameters remained unchanged between measured groups (Figure 9C). Ca^2+^/calmodulin-dependent protein kinase II expression also showed no differences between groups (Figure 9C). Interestingly, the expression of the Na^+^/Ca^2+^ exchanger (NCX) was decreased in TG compared to WT under basal and ISO-stimulated conditions, whereas no differences were detectable for both Cav1.2 and the SR Ca^2 + ^ATPase (Figure 9D).

## Discussion

Although our understanding of the structure and function of protein phosphatases, particularly as they relate to the heart and various pathophysiological states, has steadily increased over the past thirty years, we still have a limited understanding of the specific contributions made by individual regulatory subunits. This is likely due in part to an overly simplified understanding of the role these subunits play in regulating the activity of the PP2A holoenzyme, which became widespread after initial cloning and expression studies conducted in the 1990s (32, 33). However, recent advances in our approach to studying these subunits have revealed that posttranslational processes can impact not only the activity, but also the intracellular targeting and substrate specificity of the PP2A heterotrimer. Thus far, this has been demonstrated mainly for subunits belonging to the B and B' families (34–36). While early basic biochemical experiments have shown that PR72, a member of the B'' gene family, plays a role in regulating PP2A activity in response to varying Ca^2+^ concentrations (8, 9), comprehensive functional findings in both healthy and diseased hearts are still lacking. In line with our initial aim, namely to test whether the twofold increase in PR72 protein expression in human heart failure represents a compensatory mechanism to maintain contractile function, this study provides data indicating that specific 2.5-fold overexpression of PR72 in the mouse heart (1) induces a moderate cardiac hypertrophy, (2) results in increased contractile force in both whole animals and isolated cardiomyocytes, and (3) alters intracellular Ca^2+^ cycling as evidenced by increased peak amplitudes of Ca^2+^ transients, enhanced spatial spread of Ca^2+^ sparks, higher phospholamban phosphorylation and reduced NCX expression. In addition to our studies conducted on the transgenic animal model, we have also demonstrated through immunocytological, cell biological, and biochemical experiments that these effects are supported by a specific intramyocellular localization and interaction of PR72 with other partners of the PP2A heterotrimer.

An unexpected finding of this study was the observation of mild cardiac hypertrophy in TG mice that was associated with a higher mRNA expression of skeletal muscle *α*-actin. While the causes of this remain unclear and require further experimental work, such as studying the development of hypertrophy at older ages in transgenic mice, new evidence suggests a link between PR72 and the development of fibrosis. Specifically, the expression of the *PPP2R3A* gene in heterogeneous fibroblasts has been shown to initiate pulmonary fibrosis, as reported by Shi et al. (13). This process was associated with an increase in the expression of inflammatory and proliferative genes, as well as an increase in cell viability, proliferation, and migration. The effects of PR72 expression in lung fibroblasts were found to be linked to a direct effect of TGF-*β*1. The promoting effects of PR72 on proliferation can also be observed in recent tumor biology studies. For instance, high expression of the *PPP2R3A* gene was found to be directly linked to the outcome of patients with hepatocellular carcinoma (37), which correlates with promotion of glycolysis via regulation of hexokinase 1 (38). Conversely, silencing of the *PPP2R3A* gene abolished glycolysis as well as proliferation, migration, and invasion in liver tumor cells. At the same time, G1/S transition was delayed in liver tumor cell lines. However, the inhibitory effects of *PPP2R3A* silencing on cell proliferation were not only observed in liver cell tumors but also in rat primary cardiomyocytes and H9c2 cells (14). Deletion of *PPP2R3A* resulted in the inhibition of myocardial cell proliferation, cell cycle arrest in the S phase, and promotion of apoptosis. Additionally, the study identified potential candidate genes, such as *COL1A2* and *BCL6*, which interact with *PPP2R3A* and are also believed to play a role in the progression of myocardial infarction and hypertrophic cardiomyopathy (39) or in tumor development (40). But not only do the candidate genes that interact with PPP2R3A show altered expression in common heart diseases, but PR72 itself also exhibits changes in expression. For instance, *PPP2R3A* expression is reduced in a mouse model of dilated cardiomyopathy (41). *PPP2R3A* is in turn a component of the Wnt signaling pathway, which is linked to cardinal fibrosis, myocardial infarction, and heart failure (42). Other genomic studies have also demonstrated an association between the *PPP2R3A* gene and cardiomyopathy and coronary artery disease in humans (43, 44). By incorporating our data on myocardial hypertrophy in TG animals, we suggest that increased expression of PR72 initiates a gene program that may explain the structural limitations in various cardiac diseases. In this context, the transgenic PP2A-PR72 model could be a promising tool for identifying hypertrophy-associated signaling pathways.

However, it is possible that the *PPP2R3A*-associated detrimental effects on cardiac size and structure are only a secondary or even final consequence of the initial increase in PR72 expression, which likely counteracts the onset of contractility attenuation and intracellular Ca^2+^ decline in failing hearts. This would suggest, in accordance with our initial hypothesis, that the increase in PR72 expression observed in heart failure (12) represents an initial attempt to preserve contractility and Ca^2+^ homeostasis. In other words, this may come at the expense of triggering a hypertrophic gene program. Our study demonstrated at multiple levels that cardiac-specific overexpression of PR72 increased contractility in both whole animals and isolated cardiomyocytes. This was associated with a higher peak amplitude of Ca^2+^ transients. The pronounced increase in myocellular contraction compared to the relatively modest increase in the maximum amplitude of Ca^2+^ transients might suggest a higher myofilamentary Ca^2+^ sensitivity. Nevertheless, the phosphorylation status of important contractile proteins (e.g., cTnI) remained unchanged in TG. This, in turn, raises questions about the factors contributing to the increase in peak amplitude of Ca^2+^ transients, especially considering that the SR Ca^2+^ load remained unchanged in TG cardiomyocytes. To address this issue, we turned our attention to the decreased protein expression of the NCX in transgenic hearts. Partial NCX blockade, comparable to the situation in our model, by the use of specific inhibitors of NCX in healthy mice resulted in an increase in systolic Ca^2+^ transient amplitudes and a concomitant positive inotropic effect (45). Even when the *NCX1* gene was almost completely silenced in heart-specific NCX knockout mice, normal Ca^2+^ transients and an unchanged SR Ca^2+^ load were detected (46). Pott and coworkers speculated that reduction of Ca^2+^ influx by LTCC leads to an increase in excitation-contraction coupling gain (47); however, *I*_CaL_ was unchanged in our transgenic model. Because SR Ca^2+^ storage is unchanged in TG cardiomyocytes, it can be assumed that the reduced net Ca^2+^ extrusion caused by the decrease in NCX expression has direct effects on cytosolic Ca^2+^ diffusion and buffer capacity. Previous studies have shown that, on the one hand, the duration of Ca^2+^ sparks is dependent on Ca^2+^ removal from the cytoplasm by the NCX and, on the other hand, the size or spatial spread of Ca^2+^ sparks is determined by cytosolic Ca^2+^ diffusion and buffer capacity (48, 49). In the heart-specific NCX KO mice, larger and longer Ca^2+^ sparks were also evident (50). Thus, the enlargement of FDHM and FWHM in the PR72-overexpressing cardiomyocytes could be caused by a decrease in NCX-mediated Ca^2+^ removal and could further explain the increase in Ca^2+^ transient peak amplitude.

Moreover, overexpression of PR72 was associated with positive lusitropic effects in terms of cardiomyocyte relengthening and Ca^2+^ decay. Recent experiments in a mouse model with global depletion of the epidermal growth factor receptor (16) indirectly confirm our measurements. The animals exhibited a prolonged decay in Ca^2+^ transients and decreased phosphorylation of phospholamban at serine-16 and threonine-17, which was associated with the downregulation of PR72. The deteriorated cardiac phenotype was rescued by retro-orbital injection of recombinant AAV9 vectors encoding murine PR72. But how can an improved myocellular relaxation and faster decay of Ca^2+^ transients occur with PR72 overexpression?

The increased phosphorylation of phospholamban at threonine-17 could well explain the changes of both parameters in TG (51, 52). This effect seems specific to PP2A-PR72 in TG, as only TG cardiomyocytes showed a further hastening of cell relengthening and Ca^2+^ transient decay after 3 nM okadaic acid administration (53). As the expression of CaMKII was unaffected in TG hearts, these effects suggest that PR72 may inhibit phospholamban-associated PP2A activity or restrict PP2A access to its organelle-related targets, including phospholamban. This would also explain why PR72 is primarily found in the cytosol with both catalytic and structural subunits under normal conditions, as indicated by our measurements and previous studies (12, 31). Interestingly, okadaic acid had no impact on kinetic parameters in WT cells, suggesting that PP2A-PR72 targeting is specific to certain signaling pathways, transmitters, or disease states. For instance, PP2A-PR72-mediated dephosphorylation of dopamine- and cAMP-regulated phosphoprotein of 32 kDa (DARPP-32) relies on intracellular Ca^2+^ concentration (9). On the other hand, in a transgenic model of LQT2 syndrome with fatal ventricular tachyarrhythmia, increased expression of PR72 is associated with reduced PP2A activity on RyR2, resulting in increased phosphorylation of the Ca^2+^ release channel (54). In our model, however, the phosphorylation of RyR2 both at serine-2808 (PKA site) and at serine-2814 (CaMKII site) remained unchanged, indicating that the Ca^2+^ release channel is not a general substrate of PP2A-PR72.

In addition to Ca^2+^ concentration, activation of the *β*-adrenergic signaling pathway also appears to influence the substrate specificity of PP2A-PR72, as an increase in phosphorylation of MyBP-C at serine-282 in TG is observed only after administration of isoproterenol. This effect was based on a normal basal *β*-adrenergic signaling, because both cAMP levels and protein expression of the catalytic subunit of PKA were unchanged in TG hearts. The increased phosphorylation of MyBP-C in TG seems at first to be contradictory to the increase in PP activity in ISO-stimulated hearts. The latter, in turn, could be due to a mouse strain-dependent desensitization of *β*-adrenergic regulation (55). Nevertheless, irrespective of alterations in PP activity in whole-heart homogenates, exposure to catecholamines could lead to myocellular redistribution of PP2A mediated by different regulatory subunits, as shown for the regulatory B' subunit B56*α* (56). In this study, *β*-adrenergic stimulation prompted the relocation of PP2A from myofilaments to the cytosol. Consequently, changes in overall PP2A activity provide only limited insight into the activity of the enzyme at specific substrates. Future studies should aim to identify these targets of PR72 in order to not only gain a better understanding of the intracardiac regulation of PP2A-PR72 but also recognize this regulatory B'' subunit as a potential therapeutic target in various heart diseases. The development of small molecule activators of PP2A (SMAPs), which can selectively target specific PP2A holoenzymes [e.g., PP2A/B56*α*; (57, 58)] to individual Ca^2+^ regulatory or myofilament proteins leading to a normalization of disrupted phosphorylation of phosphoproteins in heart remodeling and dysfunction, demonstrates that this should not remain just a mere vision, especially for the latter goal.

In principle, genetically modified mouse models like transgenic overexpressors have been proven to be powerful tools for our understanding of molecular mechanisms in myocardium. Despite their many advantages, there are significant limitations of such studies using transgenic mice. Overexpression of a given gene in cardiomyocytes does not always result in the anticipated cardiac phenotype. For example, multiple protein expression of a gene leads to potential non-specific effects, such as ectopic expression, buffering effects, and overemphasis of certain signaling pathways. Therefore, interpretation of the significance of the study findings must be taken carefully, especially when data are extrapolated to findings in humans, as it was designed for our study.

Notwithstanding the limitations of transgenic models, our data suggest that the substantial increase in PR72 protein expression observed in heart failure may contribute to preserve contractile function. This can be deduced from the fact that elevated levels of the regulatory B'' subunit PP2A-PR72 in transgenic mice are linked to the development of mild cardiac hypertrophy and positive effects on cardiac contractility. Both the higher peak amplitude of Ca^2+^ transients and the increased spatial spread of Ca^2+^ sparks coincided with reduced expression of the NCX. The hastened relengthening of cardiomyocytes and the faster decay of Ca^2+^ transients may result from an increased phosphorylation of phospholamban at threonine-17. Experiments with okadaic acid suggest that PR72 may inhibit PP2A activity on specific substrates or restrict phosphatase access to these targets. Identifying these targets should be the focus of future research, aiming for targeted therapeutic control of cardiac output using SMAPs.

## Data Availability

The original contributions presented in the study are included in the article/**Supplementary Material**, further inquiries can be directed to the corresponding author.

## Appendix

*Cloning of expression vectors (cDNA, aMHC, RGS-6xHis)* Wildtype mouse ventricles were used to isolate mRNA using the Direct-zol RNA Miniprep kit (Zymo Research). The mRNA was then transcribed into DNA using the Transcriptor First Strand cDNA Synthesis kit (Roche), following the protocols provided by the manufacturers. To amplify the PPP2R3A/PR72 cDNA, Q5 High-Fidelity 2X Master Mix (NEB) and specific PPP2R3A/PR72 primers (forward: AAAGTAAACATTCACTTTCC; reverse: TTACAAAGCAGTGTACAAAC) were used. The amplified PPP2R3A/PR72 cDNA was integrated into a mouse cardiac α-myosin heavy chain promoter expression cassette (αMHC) to generate a construct for heart-directed over-expression. For this purpose, the PR72 cDNA was phosphorylated using T4 Polynucleotide Kinase (Thermo Fisher Scientific) and then ligated into the αMHC vector (Kirchhefer et al. 2014), which had been previously cut by Eco53ki (NEB). To obtain a His-tagged PR72 protein expression construct for mammalian cell culture, N-terminal ApaI and C-terminal XhoI restriction sites were added to the PR72 cDNA by PCR using specific primers (forward: TCTTCGGGCCCATGATGATCAAGGAAACGTCC; reverse: TCTCTTCTCGAGCTACTCTTCATCCACTGACTG). The PCR product was cloned into an RGS-6xHis-pcDNA3.1(-) vector by restriction digest with ApaI and XhoI (NEB), followed by ligation. The RGS-6xHis-pcDNA3.1(-) vector was obtained as a gift from Adam Antebi (Addgene plasmid #52534; http://n2t.net/addgene:52534; RRID: Addgene_52534; Horn et al. 2014, https://doi.org/10.1016/j.devcel.2014.01.028). All ligation reactions were performed using T4 DNA ligase (NEB). The cloned constructs were transformed into competent E. coli (RbCl) through heat-shock. Successful cloning was verified by digestion with BglII (NEB) and confirmed by sequencing (Eurofins).
