# Supplementary figures and images for: Hypercontractile cardiac phenotype in mice overexpressing the regulatory subunit PR72 of protein phosphatase 2A
Source: Front Cardiovasc Med. 2023 Oct 6;10:1239555. doi: 10.3389/fcvm.2023.1239555 (PMC10590119; doi:10.3389/fcvm.2023.1239555)

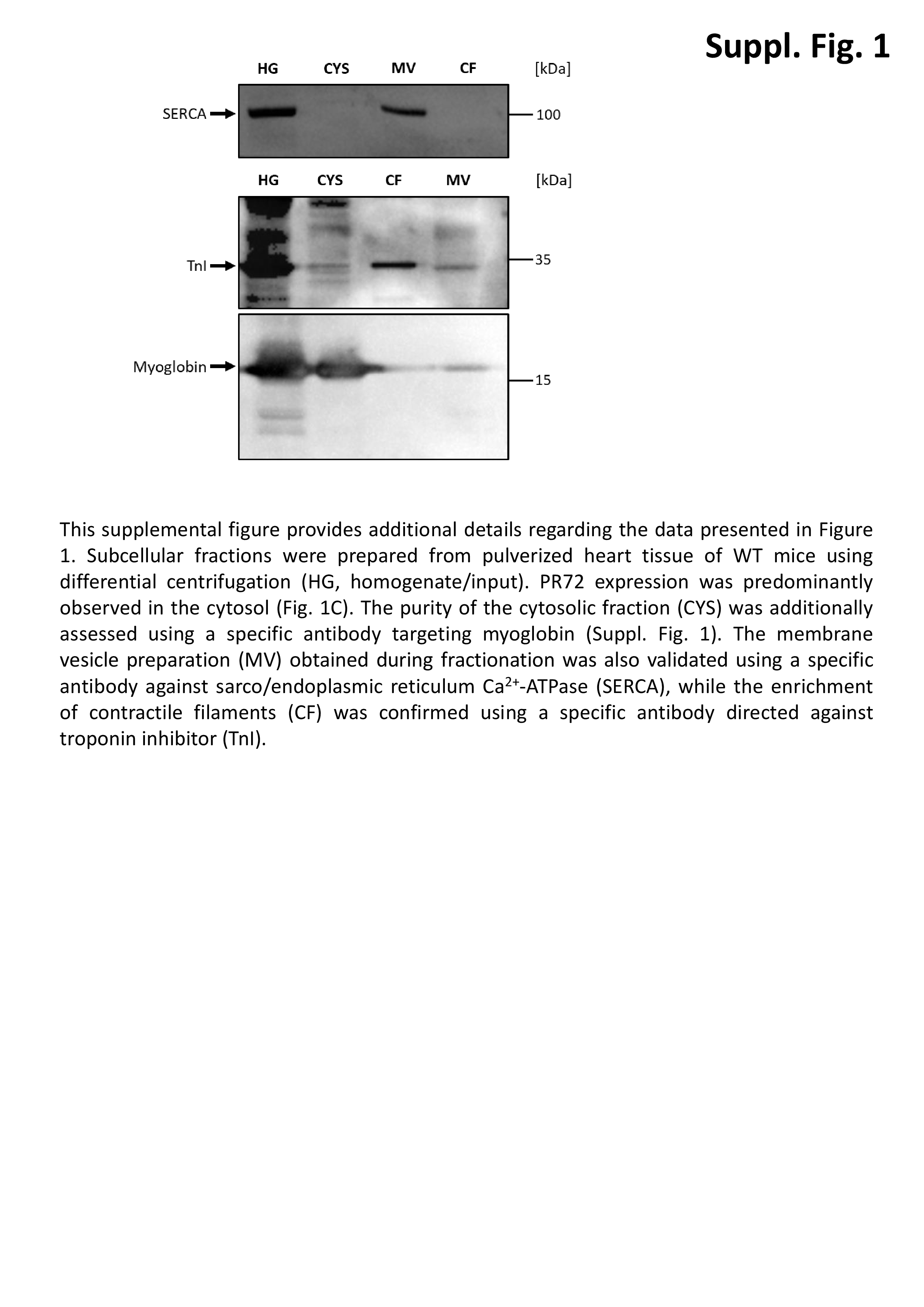

Supplement: Supplementary file 1 [file Image1.tiff]

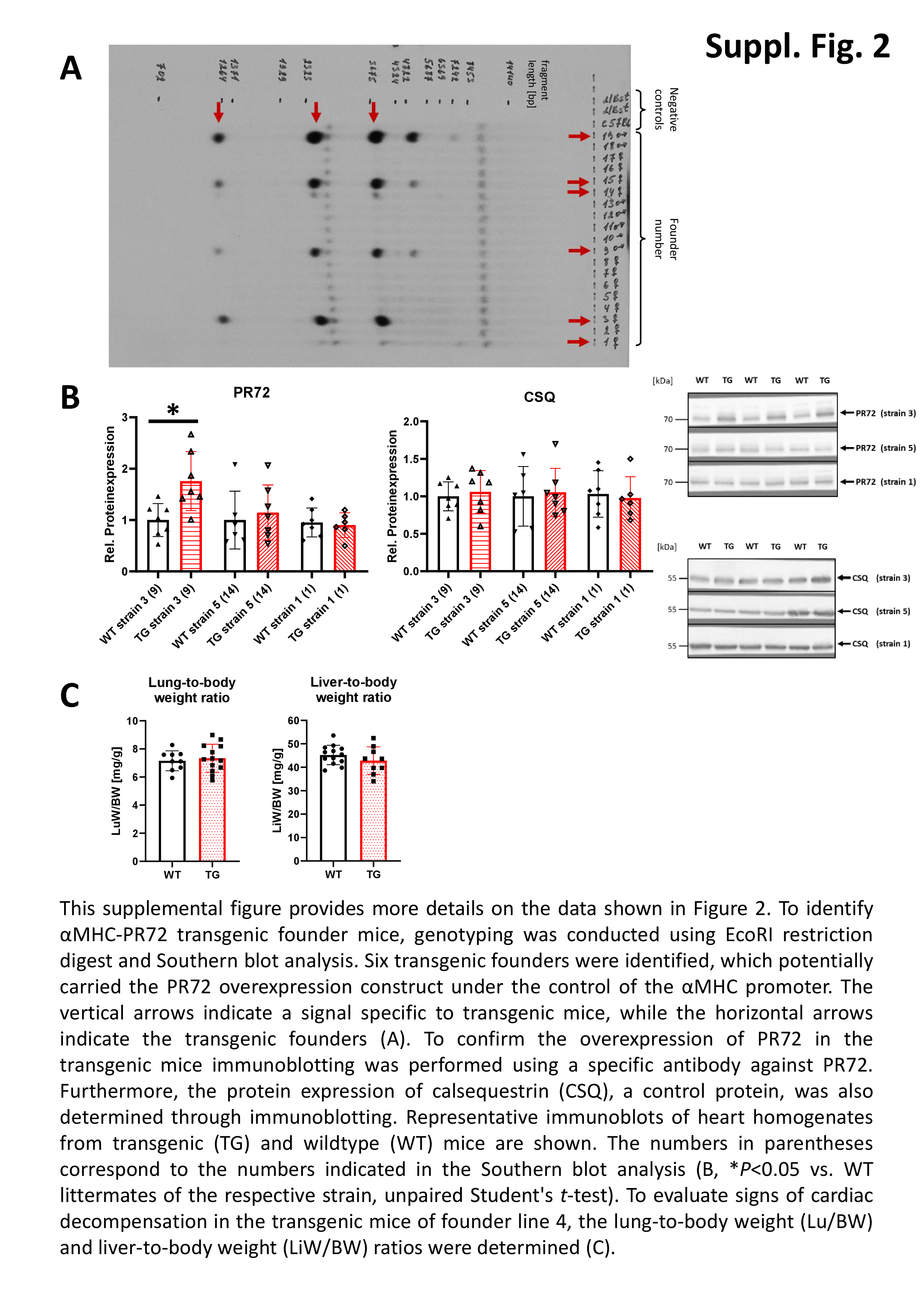

Supplement: Supplementary file 2 [file Image2.tiff]
